# Influences of Excitation on Dynamic Characteristics of Piezoelectric Micro-Jets

**DOI:** 10.3390/mi8070213

**Published:** 2017-07-05

**Authors:** Kai Li, Jun-Kao Liu, Wei-Shan Chen, Lu Zhang

**Affiliations:** 1 State Key Laboratory of Robotics and System, Harbin Institute of Technology, Harbin 150001, China; sdcxlikai@126.com (K.L.); cws@hit.edu.cn (W.-S.C.); 2 Aero Engine Corporation of China, Harbin Dongan Engine Corporation LTD, Harbin 150001, China; zhanglu916hit@163.com

**Keywords:** coupling analysis, fluid dynamic, piezoelectric micro-jet

## Abstract

Piezoelectric micro-jets are based on piezoelectric ink-jet technology and can achieve the drop-on demand requirements. A piezoelectric micro-jet which is designed for bearing lubrication is presented in this paper. In order to analyze the fluid dynamic characteristics of the piezoelectric micro-jet so as to obtain good injection performance, a direct coupling simulation method is proposed in this paper. The effects of inlet and viscous losses in the cavity are taken into account, which are close to the actual conditions in the direct coupling method. The effects of the pulse excitation parameters on the pinch-off time, tail length, velocity, and volume of the droplet are analyzed by the proposed direct coupling method. The pressure distribution inside the cavity of the micro-jet and the status of the droplet formation at different times are also given. In addition, the method is proved to be effective in predicting and analyzing the fluid dynamic characteristics of piezoelectric micro-jets by comparing the simulation results with the experimental results.

## 1. Introduction

As it can achieve drop-on-demand injection, piezoelectric micro-jet technology is widely applied in various fields, such as ejecting metal nanoparticles [1], cell printing [2,3,4], color printing [5], drug delivery [6,7,8], manufacturing [9,10,11,12,13], and sensors [14,15,16,17], etc. In addition, there are other alternate technologies to achieve drop-on-demand injection such as pyroelectric ink-jet printing [18,19,20,21,22], etc. In order to improve the injection performance, the influences of excitation parameters on fluid dynamic characteristics should be analyzed. Researchers have studied these dynamic characteristics by experiments and obtained the actual injection performance of the micro-jet [23,24,25,26,27,28,29,30,31]. Also, simulations have been used to study the characteristics of the micro-jet, as they can help to understand the outcomes of experiments and can help to pave the way to new designs. Of course, such designs should and must be checked experimentally. Many simulation projects have been carried out, but piezoelectric micro-jets with small cavities are often neglected in simulations, and only the dynamic characteristics of the nozzle part are analyzed [32,33,34,35,36,37,38]. For piezoelectric micro-jets with large cavities, such as the lubricating micro-jet presented in this paper, the influences of oil inlet and viscosity loss in the cavity cannot be neglected. In our previous study, an indirect simulation method was proposed to analyze the dynamic characteristics of the nozzle part of the piezoelectric micro-jet for lubricating, and although the results were consistent with the experimental results, the accuracy was not ideal when the quantitative analyses were carried out [39]. Therefore, a direct coupling simulation method, which takes the viscous loss and inlet effect into account, is proposed in this paper. Then, quantitatively analyses are carried out to study the influences of pulse voltage parameters on the injection performance, and the validity of this method is proved by the experiments. As some air will be trapped as bubbles in the cavity, which reduces the injection performance of the piezoelectric micro-jet [40], here the influences of bubbles are not discussed.

As the designed micro-jet is used for bearing lubrication, for a single time oil supply, the required oil volume should be met by controlling the ejected number of oil droplets. Moreover, the response time of the lubrication, which affects the timeliness of lubrication, is effected by the velocity of droplets. Thus, the volume and velocity of oil droplets are key parameters. In this paper, we obtained the volumes of the droplets under a single voltage pulse; then, we were able to determine the required number of voltage pulses for different oil volume requirements. In general, the piezoelectric micro-jet is designed to be embedded in the bearing system, and the nozzles are located between the inner and outer raceways of the bearing system. All of the ejected oil droplets will eventually be transported to the raceways with the assistance of the balls; thus, the directionality and precision of landing place are not discussed in this paper. The volume of the droplets is discussed here, as are the total volume including the volumes of satellite droplets. In order to improve the uniformity of lubrication, two nozzles are symmetrical designed. 

## 2. Working Principle

The piezoelectric micro-jet analyzed here is designed for the lubrication of a bearing system; the micro-jets are embedded in the bearing system without increasing the mass and volume of the whole system, as described in our previous work [39]. When high levels of the pulse voltages are applied to the piezoelectric vibrator, the droplets are ejected out by the positive pressure waves created in the cavity, and some air enters into the cavity from the nozzle. Then, low levels of the pulse voltages are applied, the vibrator is restored to its original state, negative pressure is created in the cavity, and the cavity is refilled by the oil from the inlet, while some of the air is squeezed out as well. Thus, we maintain the back-pressure a little higher than the atmospheric pressure by a dispenser, and the lubricating oil will not be pushed out due to its high viscosity and the small nozzle size, the back-pressure is verified in experiments (about 500 to 1000 Pa). In the simulations, the initial pressure boundary condition of the inlet is set as zero and a constant value (500 Pa) is set after one pulse excitation to simulation the function of the dispenser, while the volume fraction of the inlet is set as 1, namely, the inlet part is filled with oil.

**Figure 1:**
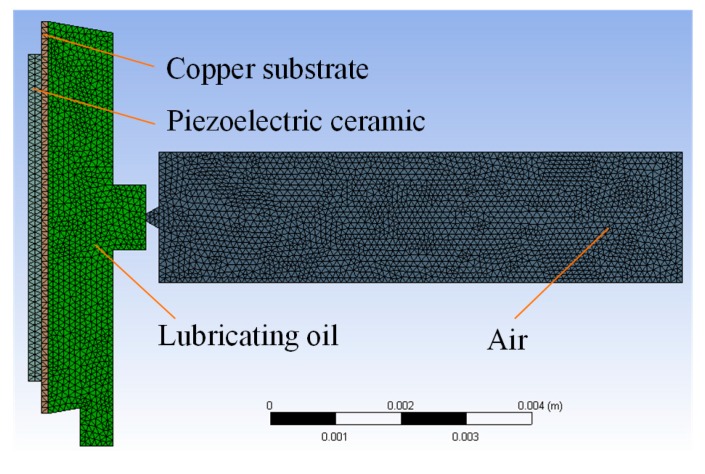
The model used in simulations.

According to the indirect coupling method proposed in our previous work, the displacements of particles on the vibrator at different positions and timepoints should be obtained first, following which the inlet velocities of the nozzle are calculated, which depend on the derived model by calculating the volume changes in the cavity. The indirect coupling method assumes that the fluid is completely incompressible, while the impact of the lubricating supply is ignored. For the direct coupling method proposed here, the velocities of particles at different positions and timepoints are applied as the velocity boundary conditions of the fluid-solid coupling interface. Moreover, the influence of the lubricating supply is taken into account, and the method can be used to simulate a compressible fluid. The model used in the simulations is shown in Figure 1; the coupling interface is between the copper substrate and the lubricating oil domain. The piezoelectric micro-jet is a central symmetrical rotating device; Figure 1 illustrates the sectional view of the device for two-dimensional simplification. In order to reduce the contact between the droplets and the outermost shell during the molding processes, the nozzle is designed as conical. Furthermore, because of the complex processing of machining the cone-shape in the shell, we selected inverse cone instead. The diameter of the nozzle is designed as 0.1 mm.

**Figure 2:**
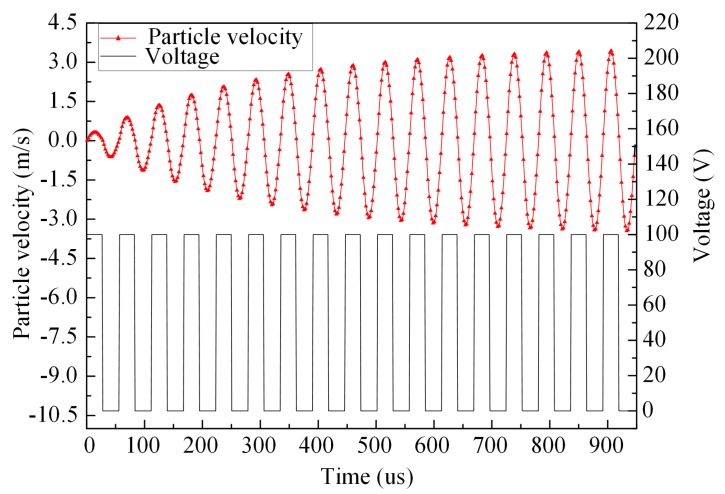
The particle velocity and its corresponding pulse voltage.

As the piezoelectric vibrator is centrally symmetrical (as shown in our two-dimensional simplification of the design), here we use *R* to represent the radial distance of the particle on the vibrator to its symmetry axis. The resonance frequency of the vibrator is largely affected by the lubricating oil due to the coupling effect. Here, the resonance frequency of the vibrator is 1.83 kHz, which is obtained by the concerned coupling effect. Therefore, the driving frequency is set as 1.83 kHz as well. The velocities of the particle (with radial distance *R* = 15 mm) at different timepoints are shown in Figure 2. As it can be seen in the figure, the vibration tends to be stable after 15 cycles. Thus, in order to simulate the fluid dynamic characteristics of the stable work of the micro-jet, the particle velocities at the steady state are chosen as the velocity inlet boundary condition of the coupling interface.

**Figure 3:**
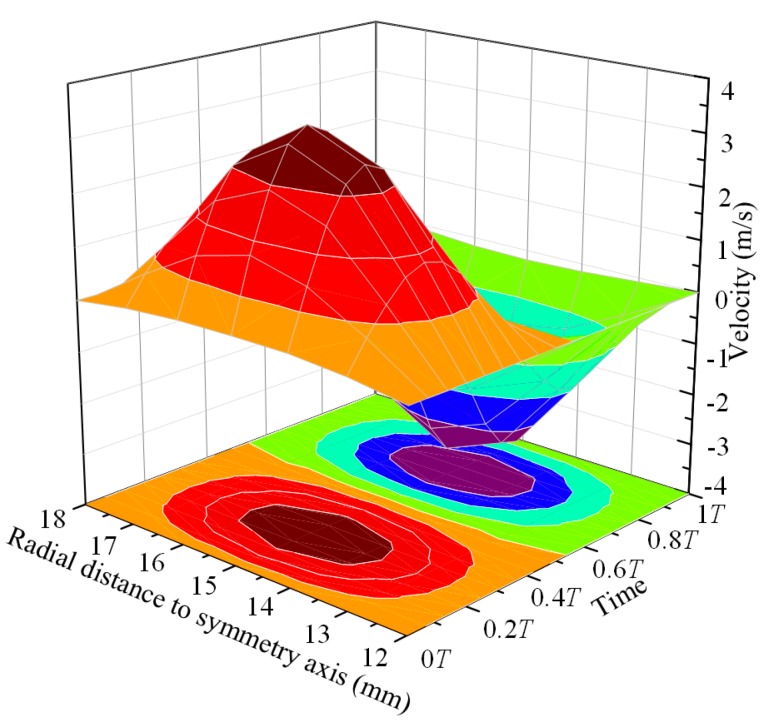
The velocities of the particles in the fluid-solid coupling interface.

The relationship between interface particle velocities and time is shown in Figure 3. As shown in the figure, the maximum particle velocity occurs at the center (radial distance *R* = 15 mm) of the interface. Due to the effect of inertia force, the particle will have a reverse speed when the pulse excitation is removed. The velocity data at different positions and timepoints is used as the velocity inlet boundary conditions of the fluid-solid coupling interface in the next two-phase flow simulations.

The fluid dynamic characteristics of the micro-jet are analyzed by using the finite element software Fluent, and the two-dimensional model of the micro-jet is shown in Figure 1. The volume of fluid (VOF) model is selected to solve the two-phase flow problem. The density and viscosity of the lubricating oil are set as 859 kg/m^3^ and 8.91 × 10^−3^ Pa∙s, respectively. The density and viscosity of the air is set as 1.225 kg/m^3^ and 1.7894 × 10^−5^ Pa∙s, respectively. The air is set as the primary phase and the continuum surface force model is selected with a constant surface tension of 0.041 N/m. The boundary condition of the fluid-solid coupling interface is set as velocity-inlet, and the velocity values at different positions depend on the particle velocity data obtained from the vibration simulations of the vibrator. The boundary condition of the oil supply inlet is set as velocity-inlet with a constant value of 0. The contact angel of the inner wall of the cavity and the outer wall of the nozzle are set as 85° and 165°, respectively. The boundary condition of the outlet in air part is set as pressure-outlet with a constant pressure value of 0.

## 3. Analysis Results

**Figure 4:**
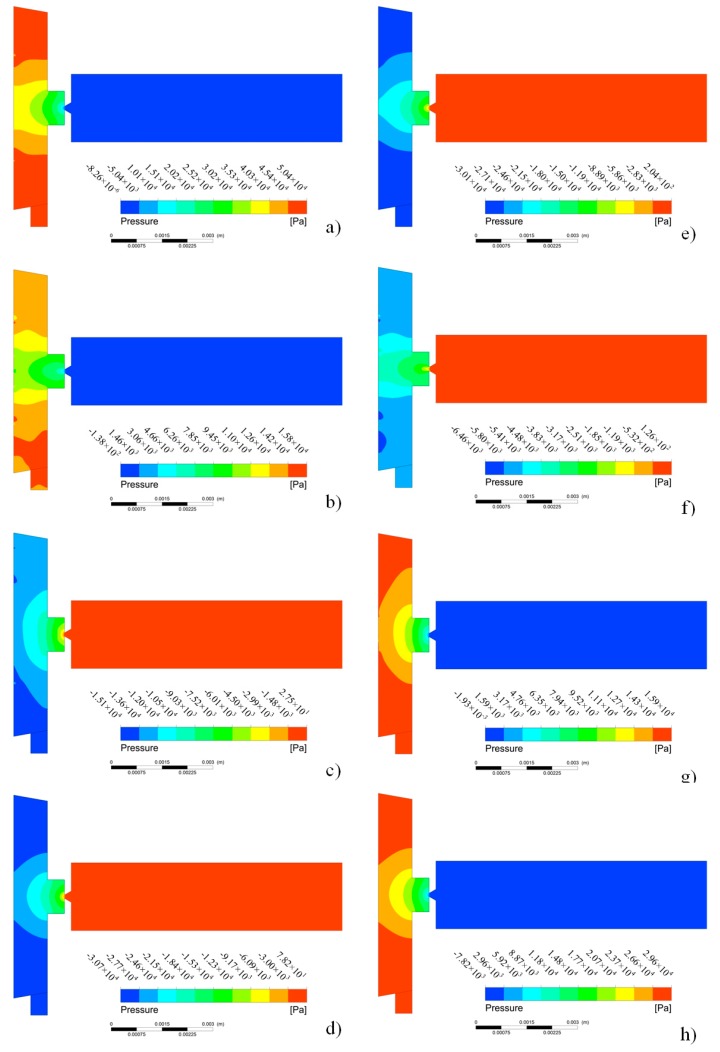
The pressure distribution at different timepoints: (**a**) 0.12*T*; (**b**) 0.25*T*; (**c**) 0.38*T*; (**d**) 0.5*T*; (**e**) 0.64*T*; (**f**) 0. 75*T*; (**g**) 0.88*T*; (**h**) 1*T*.

In order to obtain the optimal pulse voltages to improve the injection performance, the influences of pulse excitation parameters on the fluid dynamic characteristics are analyzed. When the duty ratio and amplitude of the pulse voltages are set as 0.5 and 100 V, respectively, the pressure distributions in the cavity of the micro-jet during a period are shown in Figure 4, where *T* is the period. It can be see that, with the increase of time, the pressure of the nozzle increases before 0.25*T* and then decreases after it, which is consistent with the loaded pulse voltage. After 0.5*T*, the nozzle pressure value gradually changes to negative and the absolute value reaches the maximum at nearly 0.75*T*, which is due to the effect of the inertia force of the vibrator. 

The parameters which are related to the injection performance of the micro-jet mainly include the pinch-off time (the time at which the droplet is completely ejected out of the nozzle), which reflects the response time; the tail length (the moving distance of the droplet over the pinch-off time), which determines the minimum working distance; the velocity of the droplet, which reflects the injection intensity; and the volume of the droplet, which reflects the injection precision. By the direct coupling method, the velocity and pinch-off time of the droplet can be obtained directly; however, the tail length and the volume of the droplet must be obtained through calculations. 

**Figure 5:**
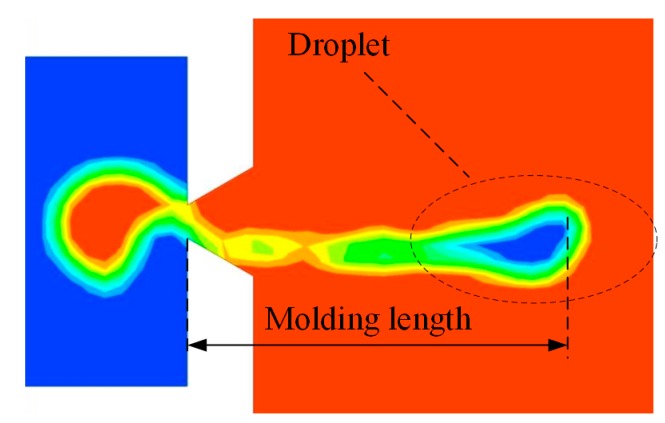
The model for the calculation of the volume and tail length of a droplet.

As shown in Figure 5, the injection status of the micro-jet at the pinch-off time can be obtained directly, then the tail length can be calculated based on the scale and measurements. The red part represents the air and the blue part represents the oil. Furthermore, as the velocity of the droplet tail is large than zero, the tail of the droplet is ejected out as well. The tail of the droplet is formed as satellite droplets. Since the model in the simulation is a two-dimensional model, the three-dimensional volume of the droplet cannot be gained directly. We establish the three-dimensional model of the droplet in relative software based on its two-dimensional model. As the volume of the liquid droplets are calculated approximately by the results of the two-dimensional model, the calculated values are larger than the actual volumes. Thus, the volume results obtained from modeling software should be multiplied by 0.613~0.684 according to our experience; here, we take 0.63.

### 3.1. The Influences of the Voltage Amplitude

**Figure 6:**
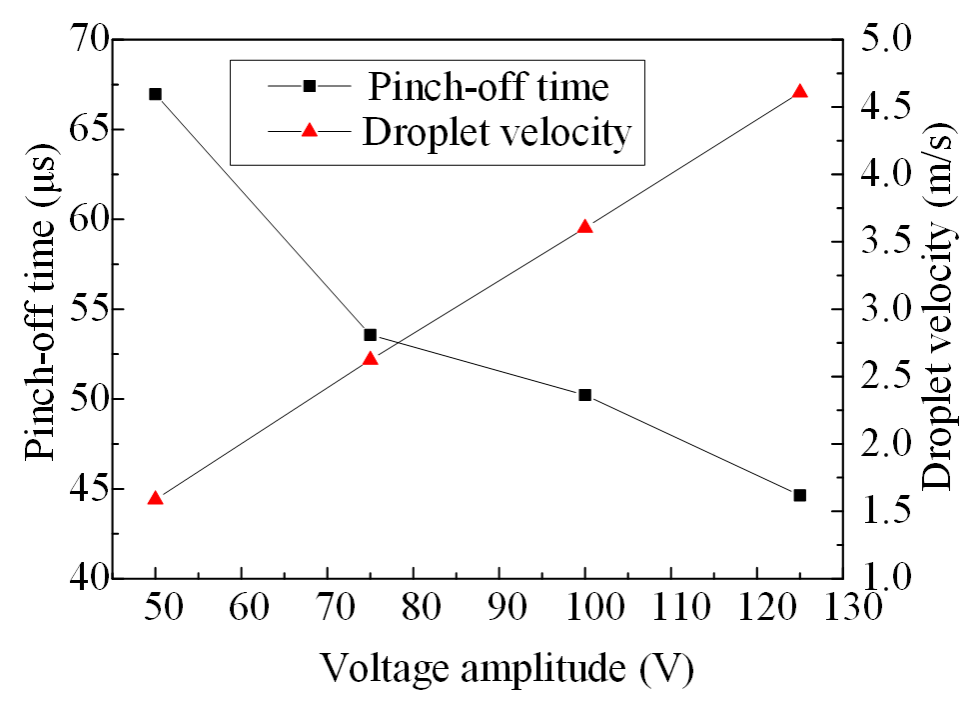
The pinch-off time and droplet velocity varies with different amplitudes of pulse voltages.

The pinch-off time and droplet velocities obtained by the direct coupling method varies with different amplitudes of pulse voltages, as shown in Figure 6. From this figure we can see that, with the increase of the voltage amplitude, the pinch-off time decreases gradually and the average change rate is −0.2976 μs∙V^−1^. The droplet velocity increases nearly lineally with the increase of the voltage amplitude, and the average change rate is 0.0403 m∙s^−1^∙V^−1^. Thus, with the increase of the voltage amplitude, both the injection intensity and the injection response speed of the micro-jet are enhanced.

**Figure 7:**
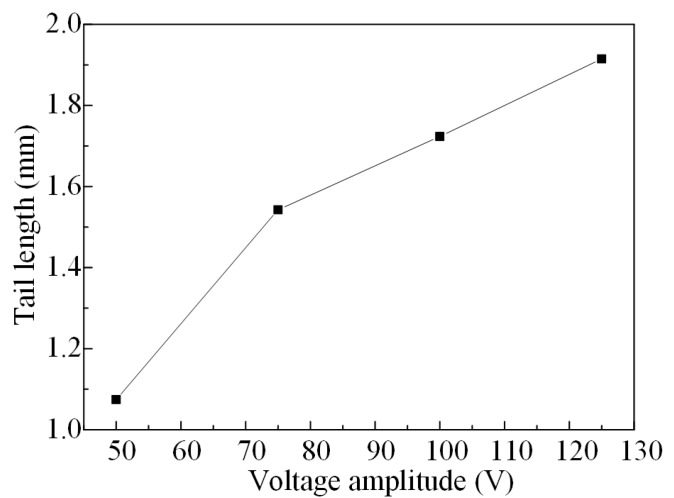
The tail length varies with different voltage amplitudes.

The tail length varies with different voltage amplitudes which, as obtained by the direct coupling method, are shown in Figure 7. From this figure, it can be seen that the tail length increases along with the increase of the voltage amplitude, and the average change rate is 0.0107 mm∙V^−1^. 

**Figure 8:**
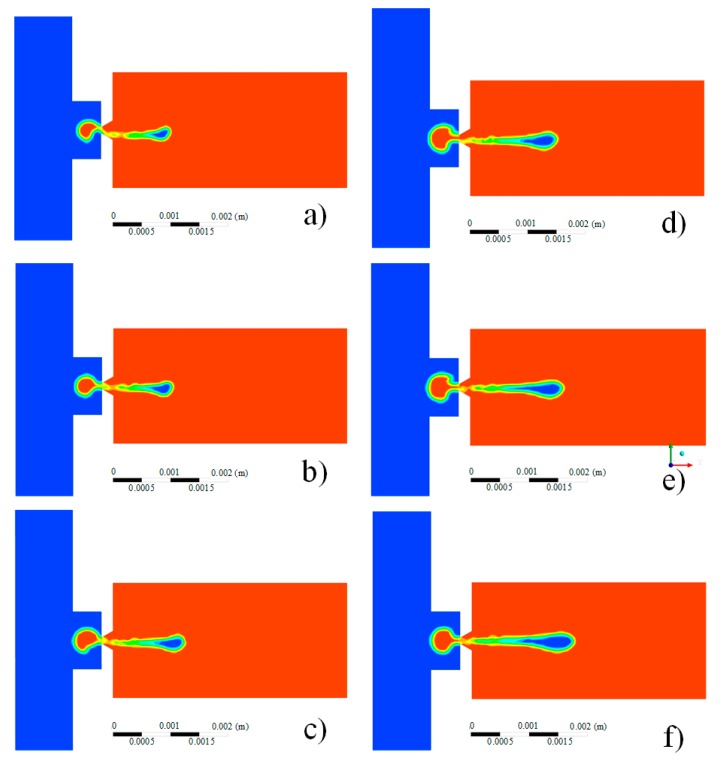
The injection status varies with different voltage amplitudes: (**a**) 50 V; (**b**) 60 V; (**c**) 75 V; (**d**) 90 V; (**e**) 100 V; (**f**) 125 V.

The injection status varies with different voltage amplitudes, which are obtained by the method of direct coupling, as shown in Figure 8. It can be seen that, when the voltage amplitude is relatively low, the ejected droplets are not symmetrical. The reason for this is that the effect of the oil supply inlet is taken into account in the direct coupling method, which results in an unsymmetrical pressure distribution at the nozzle. Furthermore, with the increase of the voltage amplitude, the effect is faded gradually. 

### 3.2. The Influence of the Duty Ratio

**Figure 9:**
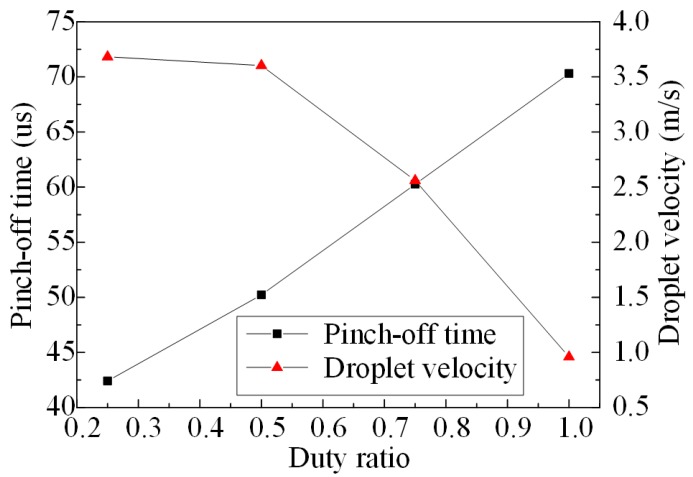
The pinch-off time and droplet velocity vary with different duty ratios.

Another important parameter of the pulse voltage is the duty ratio; the pinch-off time and droplet velocity vary with different duty ratios obtained from the direct coupling method, as shown in Figure 9. From this figure we can see that, along with the increase of the duty ratio, the pinch-off time increases nearly linearly and the droplet velocity decreases. The change rate of the pinch-off time and droplet velocity are 37.2 μs and −5.291 m/s, respectively. Therefore, with the increase of the duty ratio, both the injection intensity and the injection response speed of the micro-jet are weakened.

**Figure 10:**
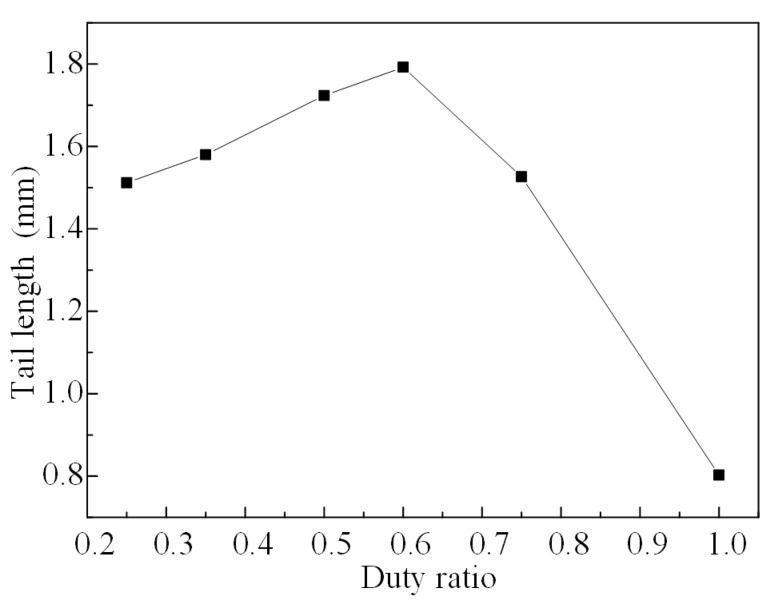
The tail length varies with different duty ratios.

With the increase of the duty ratio, the change curve of the tail length is obtained by the direct coupling method, as shown in Figure 10. From this figure we can see that, according to the results, the tail length increases before the turning point (duty ratio *α* = 0.6) and then decreases. The reason for this is that, according to the directing coupling method, with the increase of the duty ratio, the energy dissipation increases after the turning point (duty ratio *α* = 0.6) and the droplets are molded after a shorter moving distance as a result of the effect of the asymmetrical pressure at the nozzle.

**Figure 11:**
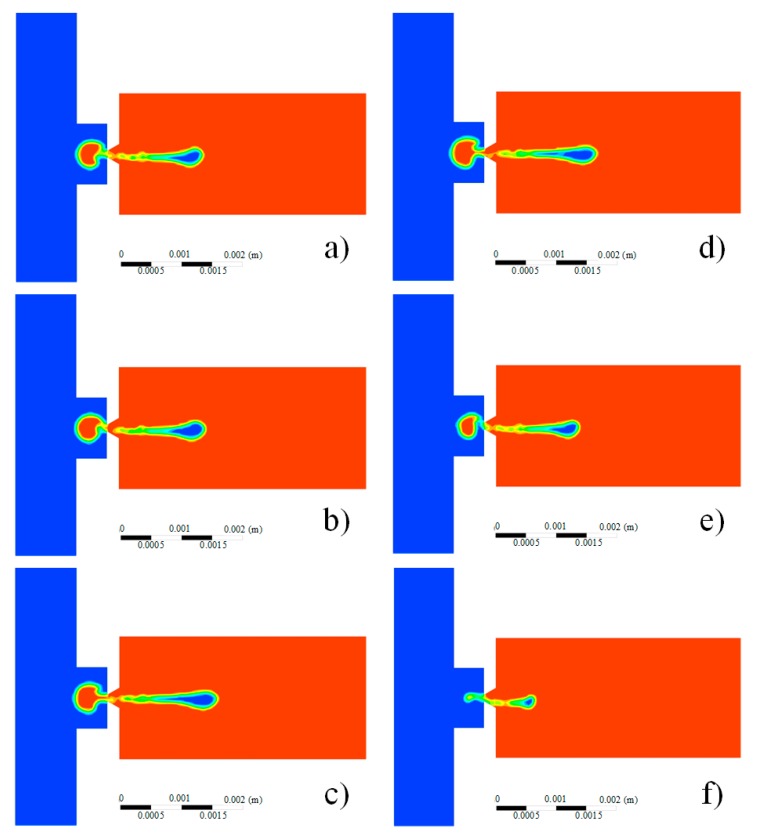
The injection status varies with different duty ratios: (**a**) *α* = 0.25; (**b**) *α* = 0.35; (**c**) *α* = 0.5; (**d**) *α* = 0.6; (**e**) *α* = 0.75; (**f**) *α* = 0.99.

As shown in Figure 11, with the increase of the duty ratio, the injection intensity increases before the turning point (duty ratio *α* = 0.6) and then decreases after it. The reason for this is that the energy loss created in the cavity, which is related to the viscosity of the lubricating oil, increases with the increase of the duty ratio.

### 3.3. Comparison with the Experiment Results

**Figure 12:**
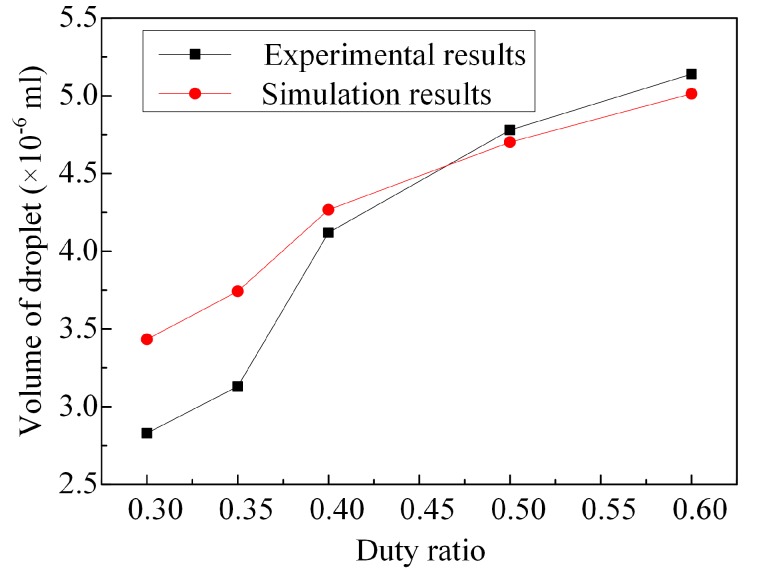
The droplet volume varies with different duty ratios.

The experimental platform and method are described in our previous work [39]. The experimental platform mainly consists of a waveform generator, digital storage oscilloscope, and power amplifier. The droplet volume varies with different duty ratios, which are obtained by experiments and simulations. As shown in Figure 12, we can see that with the increase of the duty ratio, the droplet volume increases gradually (when the duty ratio *α* < 0.6). The results of the simulation are consonant with the experimental results, and the errors are acceptable for quantitative analyses.

## 4. Conclusions

A direct coupling method which is used to simulate the fluid dynamic characteristic of the piezoelectric micro-jet is proposed in this paper, and the model built according to this method is closer to the actual structure of the micro-jet. The effect of the oil supply inlet is taken into account in the direct coupling method, and it has been proven that this effect cannot be ignored when analyzing the influence of the voltage parameters on the injection performance. The change rate of the pinch-off time, droplet velocity, and tail length are given, which can be used as the references for adjusting the injection performance by modifying the parameters of pulse voltages. Comparing the results obtained from the direct coupling method with the experimental data, it is demonstrated that the direct coupling method proves to be an effective and feasible method to quantitatively simulate the fluid dynamic characteristics of the micro-jet.

In future research, we will carry out three-dimensional simulations, as the volume of droplets can be obtained directly from the results of a three-dimensional simulation analysis, and the model is closer to reality, namely, the simulations will be more accurate.
